# 3-[2-(5*H*-Indolo[2,3-*b*]quinoxalin-5-yl)eth­yl]-1,3-oxazolidin-2-one

**DOI:** 10.1107/S1600536810033222

**Published:** 2010-08-28

**Authors:** Abdussalam Alsubari, Rachid Bouhfid, Hafid Zouihri, El Mokhtar Essassi, Seik Weng Ng

**Affiliations:** a Laboratoire de Chimie Organique Hétérocyclique, Pôle de Compétences Pharmacochimie, Université Mohammed V-Agdal, BP 1014 Avenue Ibn Batout, Rabat, Morocco; b CNRST Division UATRS, Angle Allal Fassi/FAR, BP 8027 Hay Riad, Rabat, Morocco; c Department of Chemistry, University of Malaya, 50603 Kuala Lumpur, Malaysia

## Abstract

The title compound, C_19_H_16_N_4_O_2_, has an almost planar fused *N*-heterocyclic system (r.m.s. deviation = 0.031 Å) and an almost planar five-membered 1,3-oxazolidine ring (r.m.s. deviation = 0.015 Å) at the ends of an ethyl­ene chain [N—C—C—N torsion angle = −65.6 (2)°]. The ring systems are inclined at 38.1 (1)° to one another.

## Related literature

For background to this class of oxindole derivatives, see: Alsubari *et al.* (2009 ▶). For a related structure, see: Alsubari *et al.* (2010 ▶)
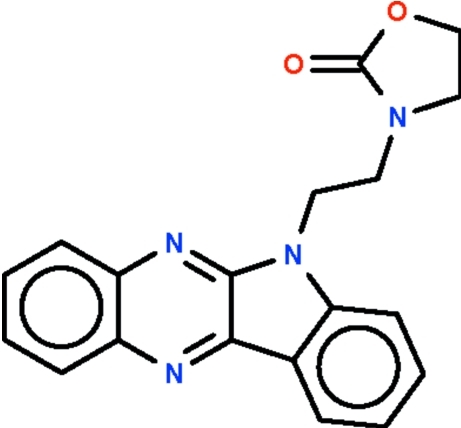


## Experimental

### 

#### Crystal data

C_19_H_16_N_4_O_2_
*M* *_r_* = 332.36
Monoclinic, 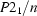

*a* = 14.5565 (4) Å
*b* = 5.8993 (2) Å
*c* = 18.6434 (6) Å
β = 92.393 (2)°
*V* = 1599.57 (9) Å^3^
*Z* = 4
Mo *K*α radiation
μ = 0.09 mm^−1^
*T* = 293 K
0.37 × 0.18 × 0.17 mm

#### Data collection

Bruker X8 APEXII diffractometer
19296 measured reflections
3216 independent reflections
1897 reflections with *I* > 2σ(*I*)
*R*_int_ = 0.054

#### Refinement

*R*[*F* ^2^ > 2σ(*F* ^2^)] = 0.044
*wR*(*F* ^2^) = 0.152
*S* = 0.95
3216 reflections
226 parameters
H-atom parameters constrained
Δρ_max_ = 0.22 e Å^−3^
Δρ_min_ = −0.15 e Å^−3^

### 

Data collection: *APEX2* (Bruker, 2008 ▶); cell refinement: *SAINT* (Bruker, 2008 ▶); data reduction: *SAINT*; program(s) used to solve structure: *SHELXS97* (Sheldrick, 2008 ▶); program(s) used to refine structure: *SHELXL97* (Sheldrick, 2008 ▶); molecular graphics: *X-SEED* (Barbour, 2001 ▶); software used to prepare material for publication: *publCIF* (Westrip, 2010 ▶).

## Supplementary Material

Crystal structure: contains datablocks global, I. DOI: 10.1107/S1600536810033222/fl2310sup1.cif

Structure factors: contains datablocks I. DOI: 10.1107/S1600536810033222/fl2310Isup2.hkl

Additional supplementary materials:  crystallographic information; 3D view; checkCIF report

## Figures and Tables

**Fig. 1.**
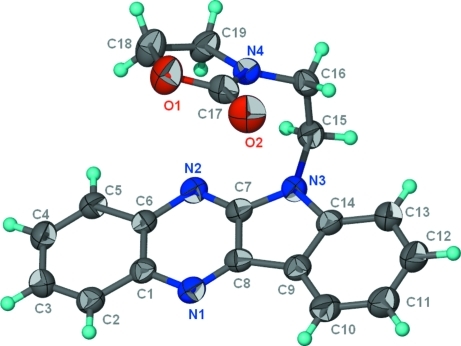
Thermal ellipsoid plot of the title compound at the 50% probability level; hydrogen atoms are drawn as spheres of arbitrary radius.

**Fig. 2.**
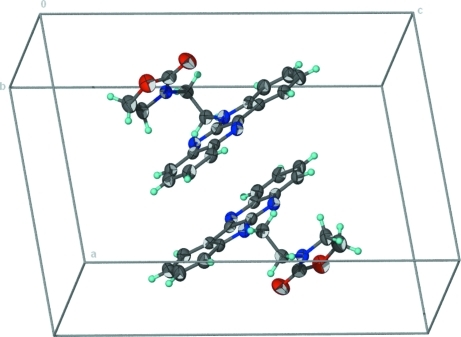
Two molecules disposed about a center-of-inversion.

## supplementary crystallographic information

### Comment

The synthesis of new oxindole derivatives having an oxazolindin-2-one unit has been detailed in a recent report (Alsubari *et al.*, 2009). Among related compounds whose structures have been determined is 3-[2-(2,3-dioxoindolin-1-yl)ethyl]-1,3-oxazolidin-2-one (Alsubari *et al.*, 2010) in which the oxazolidinyl ring has an envelope conformation with the methylene C atom bonded to the N atom as the flap. The –CH_2_–CH_2_– connecting this ring to the other fused-ring system has its substituents in a *gauche* conformation [torsion angle = 62.7 (2)°]. In the tite compound (Scheme I, Fig. 1), the oxazolidinyl ring is planar (rms 0.015 Å), and there is no indication of any disorder in the ethylene portion of the ring. The fused *N*-heterocyclic system (rms 0.031 Å) is also planar and the two ring systems are inclined at 38.1 (1)° to one another. . The fused-rings are not stacked directly over one another, however, the distance between two inversion-related fused ring systems is only 3.4 Å (Fig. 2).

### Experimental

1-(2-(2-Oxoxazolidin-3-yl)ethyl)indoline-2,3-dione (0.5 g, 3.84 mmole) and *o*-phenylenediamine (0.41 g, 3.84 mmole) were heated in xylene (30 ml) and refluxed for twelve hours. The solvent was then removed under reduced pressure and the residue recrystallized from ethanol to afford the title compound as yellow crystals.

### Refinement

Carbon-bound H-atoms were placed in calculated positions (C–H 0.93–0.97 Å) and were included in the refinement in the riding model approximation, with *U*(H) set to 1.2*U*_eq_(C).

### Crystal data

**Table:**

C_19_H_16_N_4_O_2_	*F*(000) = 696
*M**_r_* = 332.36	*D*_x_ = 1.380 Mg m^−^^3^
Monoclinic, *P*2_1_/*n*	Mo *K*α radiation, λ = 0.71073 Å
Hall symbol: -P 2yn	Cell parameters from 2575 reflections
*a* = 14.5565 (4) Å	θ = 2.8–21.3°
*b* = 5.8993 (2) Å	µ = 0.09 mm^−^^1^
*c* = 18.6434 (6) Å	*T* = 293 K
β = 92.393 (2)°	Prism, yellow
*V* = 1599.57 (9) Å^3^	0.37 × 0.18 × 0.17 mm
*Z* = 4	

### Data collection

**Table:**

Bruker X8 APEXII diffractometer	1897 reflections with *I* > 2σ(*I*)
Radiation source: fine-focus sealed tube	*R*_int_ = 0.054
graphite	θ_max_ = 26.3°, θ_min_ = 2.8°
φ and ω scans	*h* = −18→18
19296 measured reflections	*k* = −7→7
3216 independent reflections	*l* = −23→23

### Refinement

**Table:**

Refinement on *F*^2^	Primary atom site location: structure-invariant direct methods
Least-squares matrix: full	Secondary atom site location: difference Fourier map
*R*[*F*^2^ > 2σ(*F*^2^)] = 0.044	Hydrogen site location: inferred from neighbouring sites
*wR*(*F*^2^) = 0.152	H-atom parameters constrained
*S* = 0.95	*w* = 1/[σ^2^(*F*_o_^2^) + (0.0947*P*)^2^] where *P* = (*F*_o_^2^ + 2*F*_c_^2^)/3
3216 reflections	(Δ/σ)_max_ = 0.001
226 parameters	Δρ_max_ = 0.22 e Å^−^^3^
0 restraints	Δρ_min_ = −0.15 e Å^−^^3^

### Fractional atomic coordinates and isotropic or equivalent isotropic displacement parameters (Å^2^)

**Table:**

	*x*	*y*	*z*	*U*_iso_*/*U*_eq_	
O1	0.14976 (14)	0.4457 (3)	0.30554 (10)	0.0756 (6)	
O2	0.09479 (13)	0.3291 (3)	0.41022 (10)	0.0765 (6)	
N1	0.38685 (12)	0.2701 (3)	0.50246 (9)	0.0499 (5)	
N2	0.32451 (12)	0.6823 (3)	0.43058 (9)	0.0493 (5)	
N3	0.21587 (12)	0.6899 (3)	0.52289 (9)	0.0491 (5)	
N4	0.10754 (12)	0.7065 (3)	0.38211 (9)	0.0449 (4)	
C1	0.42821 (14)	0.3536 (4)	0.44272 (11)	0.0455 (5)	
C2	0.50363 (16)	0.2364 (4)	0.41630 (12)	0.0536 (6)	
H2	0.5235	0.1027	0.4383	0.064*	
C3	0.54794 (16)	0.3169 (4)	0.35861 (12)	0.0571 (6)	
H3	0.5980	0.2382	0.3418	0.069*	
C4	0.51867 (16)	0.5176 (4)	0.32443 (12)	0.0573 (6)	
H4	0.5496	0.5713	0.2852	0.069*	
C5	0.44540 (15)	0.6342 (4)	0.34824 (12)	0.0519 (6)	
H5	0.4262	0.7664	0.3249	0.062*	
C6	0.39828 (14)	0.5568 (4)	0.40790 (10)	0.0434 (5)	
C7	0.28851 (14)	0.5993 (4)	0.48773 (11)	0.0448 (5)	
C8	0.31888 (14)	0.3950 (4)	0.52400 (11)	0.0446 (5)	
C9	0.26054 (15)	0.3687 (4)	0.58425 (11)	0.0477 (5)	
C10	0.25656 (16)	0.2117 (4)	0.64019 (12)	0.0593 (6)	
H10	0.2985	0.0928	0.6437	0.071*	
C11	0.19041 (17)	0.2347 (5)	0.68979 (14)	0.0693 (7)	
H11	0.1874	0.1309	0.7272	0.083*	
C12	0.12790 (18)	0.4118 (5)	0.68458 (13)	0.0713 (8)	
H12	0.0829	0.4229	0.7184	0.086*	
C13	0.13042 (17)	0.5719 (5)	0.63082 (12)	0.0624 (7)	
H13	0.0884	0.6907	0.6282	0.075*	
C14	0.19776 (15)	0.5498 (4)	0.58088 (11)	0.0480 (6)	
C15	0.15862 (15)	0.8786 (4)	0.49739 (11)	0.0505 (6)	
H15A	0.1961	0.9839	0.4714	0.061*	
H15B	0.1353	0.9582	0.5384	0.061*	
C16	0.07797 (15)	0.8028 (4)	0.44871 (12)	0.0483 (6)	
H16A	0.0424	0.6908	0.4737	0.058*	
H16B	0.0384	0.9317	0.4381	0.058*	
C17	0.11498 (16)	0.4834 (4)	0.37101 (13)	0.0539 (6)	
C18	0.1696 (2)	0.6593 (5)	0.27265 (14)	0.0742 (8)	
H18A	0.1341	0.6763	0.2278	0.089*	
H18B	0.2344	0.6699	0.2630	0.089*	
C19	0.14339 (19)	0.8400 (4)	0.32542 (12)	0.0616 (7)	
H19A	0.1965	0.9276	0.3421	0.074*	
H19B	0.0971	0.9416	0.3046	0.074*	

### Atomic displacement parameters (Å^2^)

**Table:**

	*U*^11^	*U*^22^	*U*^33^	*U*^12^	*U*^13^	*U*^23^
O1	0.1126 (15)	0.0451 (11)	0.0699 (11)	0.0048 (9)	0.0125 (11)	−0.0150 (8)
O2	0.0993 (14)	0.0362 (10)	0.0947 (14)	−0.0026 (9)	0.0106 (11)	0.0101 (9)
N1	0.0567 (11)	0.0489 (11)	0.0437 (10)	0.0008 (9)	−0.0010 (9)	0.0018 (8)
N2	0.0515 (11)	0.0515 (11)	0.0446 (10)	0.0010 (9)	0.0001 (9)	0.0027 (8)
N3	0.0496 (11)	0.0537 (12)	0.0442 (10)	0.0081 (9)	0.0031 (9)	0.0015 (8)
N4	0.0569 (11)	0.0318 (10)	0.0459 (10)	0.0031 (8)	−0.0002 (8)	−0.0013 (8)
C1	0.0486 (12)	0.0489 (13)	0.0387 (11)	−0.0020 (10)	−0.0038 (10)	−0.0051 (9)
C2	0.0602 (14)	0.0534 (14)	0.0467 (13)	0.0070 (11)	−0.0034 (11)	−0.0031 (11)
C3	0.0562 (14)	0.0647 (16)	0.0507 (14)	0.0115 (12)	0.0040 (11)	−0.0073 (12)
C4	0.0571 (15)	0.0726 (17)	0.0426 (12)	−0.0010 (13)	0.0062 (11)	−0.0009 (12)
C5	0.0539 (13)	0.0558 (15)	0.0460 (12)	0.0022 (11)	0.0012 (11)	0.0057 (11)
C6	0.0434 (12)	0.0499 (13)	0.0366 (11)	0.0014 (10)	−0.0034 (9)	−0.0038 (9)
C7	0.0471 (12)	0.0466 (13)	0.0401 (11)	−0.0011 (10)	−0.0041 (10)	−0.0004 (10)
C8	0.0462 (12)	0.0438 (13)	0.0430 (11)	−0.0005 (10)	−0.0072 (10)	−0.0009 (10)
C9	0.0466 (12)	0.0526 (14)	0.0436 (12)	−0.0055 (10)	−0.0022 (10)	0.0002 (10)
C10	0.0585 (14)	0.0619 (16)	0.0573 (15)	−0.0005 (12)	−0.0025 (12)	0.0096 (12)
C11	0.0661 (16)	0.083 (2)	0.0587 (16)	−0.0056 (15)	0.0070 (13)	0.0199 (14)
C12	0.0602 (16)	0.102 (2)	0.0523 (14)	−0.0002 (16)	0.0111 (12)	0.0145 (15)
C13	0.0573 (15)	0.0779 (18)	0.0524 (14)	0.0068 (13)	0.0057 (12)	0.0027 (13)
C14	0.0483 (13)	0.0553 (14)	0.0401 (11)	−0.0023 (10)	−0.0031 (10)	0.0013 (10)
C15	0.0596 (14)	0.0442 (13)	0.0479 (13)	0.0055 (10)	0.0024 (11)	−0.0064 (10)
C16	0.0494 (12)	0.0401 (13)	0.0558 (13)	0.0085 (10)	0.0059 (10)	0.0024 (10)
C17	0.0604 (15)	0.0371 (13)	0.0639 (15)	0.0037 (10)	−0.0018 (12)	−0.0045 (11)
C18	0.104 (2)	0.0604 (18)	0.0588 (16)	−0.0010 (14)	0.0136 (15)	−0.0080 (12)
C19	0.0917 (18)	0.0481 (15)	0.0453 (13)	0.0035 (13)	0.0039 (12)	0.0037 (10)

### Geometric parameters (Å, °)

**Table:**

O1—C17	1.359 (3)	C7—C8	1.442 (3)
O1—C18	1.436 (3)	C8—C9	1.445 (3)
O2—C17	1.211 (3)	C9—C10	1.398 (3)
N1—C8	1.310 (3)	C9—C14	1.406 (3)
N1—C1	1.379 (3)	C10—C11	1.369 (3)
N2—C7	1.302 (3)	C10—H10	0.9300
N2—C6	1.385 (3)	C11—C12	1.386 (4)
N3—C7	1.375 (3)	C11—H11	0.9300
N3—C14	1.395 (3)	C12—C13	1.379 (3)
N3—C15	1.458 (3)	C12—H12	0.9300
N4—C17	1.338 (3)	C13—C14	1.386 (3)
N4—C19	1.434 (3)	C13—H13	0.9300
N4—C16	1.447 (3)	C15—C16	1.521 (3)
C1—C2	1.404 (3)	C15—H15A	0.9700
C1—C6	1.423 (3)	C15—H15B	0.9700
C2—C3	1.362 (3)	C16—H16A	0.9700
C2—H2	0.9300	C16—H16B	0.9700
C3—C4	1.402 (3)	C18—C19	1.510 (3)
C3—H3	0.9300	C18—H18A	0.9700
C4—C5	1.359 (3)	C18—H18B	0.9700
C4—H4	0.9300	C19—H19A	0.9700
C5—C6	1.407 (3)	C19—H19B	0.9700
C5—H5	0.9300		
			
C17—O1—C18	109.19 (18)	C10—C11—C12	120.3 (2)
C8—N1—C1	114.03 (19)	C10—C11—H11	119.8
C7—N2—C6	113.11 (18)	C12—C11—H11	119.8
C7—N3—C14	108.24 (18)	C13—C12—C11	122.0 (2)
C7—N3—C15	125.57 (18)	C13—C12—H12	119.0
C14—N3—C15	125.53 (18)	C11—C12—H12	119.0
C17—N4—C19	113.09 (19)	C12—C13—C14	117.7 (2)
C17—N4—C16	123.23 (19)	C12—C13—H13	121.1
C19—N4—C16	123.19 (18)	C14—C13—H13	121.1
N1—C1—C2	118.8 (2)	C13—C14—N3	128.8 (2)
N1—C1—C6	122.26 (19)	C13—C14—C9	121.2 (2)
C2—C1—C6	118.94 (19)	N3—C14—C9	109.98 (19)
C3—C2—C1	120.5 (2)	N3—C15—C16	112.74 (18)
C3—C2—H2	119.7	N3—C15—H15A	109.0
C1—C2—H2	119.7	C16—C15—H15A	109.0
C2—C3—C4	120.5 (2)	N3—C15—H15B	109.0
C2—C3—H3	119.7	C16—C15—H15B	109.0
C4—C3—H3	119.7	H15A—C15—H15B	107.8
C5—C4—C3	120.4 (2)	N4—C16—C15	112.19 (17)
C5—C4—H4	119.8	N4—C16—H16A	109.2
C3—C4—H4	119.8	C15—C16—H16A	109.2
C4—C5—C6	120.6 (2)	N4—C16—H16B	109.2
C4—C5—H5	119.7	C15—C16—H16B	109.2
C6—C5—H5	119.7	H16A—C16—H16B	107.9
N2—C6—C5	118.5 (2)	O2—C17—N4	128.5 (2)
N2—C6—C1	122.53 (18)	O2—C17—O1	121.9 (2)
C5—C6—C1	118.95 (19)	N4—C17—O1	109.6 (2)
N2—C7—N3	126.0 (2)	O1—C18—C19	106.30 (19)
N2—C7—C8	124.87 (19)	O1—C18—H18A	110.5
N3—C7—C8	109.16 (18)	C19—C18—H18A	110.5
N1—C8—C7	123.16 (19)	O1—C18—H18B	110.5
N1—C8—C9	130.8 (2)	C19—C18—H18B	110.5
C7—C8—C9	106.00 (18)	H18A—C18—H18B	108.7
C10—C9—C14	119.3 (2)	N4—C19—C18	101.7 (2)
C10—C9—C8	134.1 (2)	N4—C19—H19A	111.4
C14—C9—C8	106.60 (18)	C18—C19—H19A	111.4
C11—C10—C9	119.4 (2)	N4—C19—H19B	111.4
C11—C10—H10	120.3	C18—C19—H19B	111.4
C9—C10—H10	120.3	H19A—C19—H19B	109.3
			
C8—N1—C1—C2	178.18 (19)	C14—C9—C10—C11	1.3 (3)
C8—N1—C1—C6	−0.5 (3)	C8—C9—C10—C11	−179.5 (2)
N1—C1—C2—C3	−178.2 (2)	C9—C10—C11—C12	0.0 (4)
C6—C1—C2—C3	0.6 (3)	C10—C11—C12—C13	−1.0 (4)
C1—C2—C3—C4	−0.3 (3)	C11—C12—C13—C14	0.6 (4)
C2—C3—C4—C5	−0.2 (4)	C12—C13—C14—N3	−178.6 (2)
C3—C4—C5—C6	0.6 (3)	C12—C13—C14—C9	0.9 (3)
C7—N2—C6—C5	−177.54 (18)	C7—N3—C14—C13	−179.2 (2)
C7—N2—C6—C1	2.3 (3)	C15—N3—C14—C13	−8.2 (4)
C4—C5—C6—N2	179.5 (2)	C7—N3—C14—C9	1.2 (2)
C4—C5—C6—C1	−0.4 (3)	C15—N3—C14—C9	172.27 (19)
N1—C1—C6—N2	−1.3 (3)	C10—C9—C14—C13	−1.8 (3)
C2—C1—C6—N2	179.99 (19)	C8—C9—C14—C13	178.8 (2)
N1—C1—C6—C5	178.46 (19)	C10—C9—C14—N3	177.76 (19)
C2—C1—C6—C5	−0.2 (3)	C8—C9—C14—N3	−1.6 (2)
C6—N2—C7—N3	178.79 (19)	C7—N3—C15—C16	87.4 (3)
C6—N2—C7—C8	−1.6 (3)	C14—N3—C15—C16	−82.2 (2)
C14—N3—C7—N2	179.42 (19)	C17—N4—C16—C15	98.1 (2)
C15—N3—C7—N2	8.3 (3)	C19—N4—C16—C15	−73.2 (3)
C14—N3—C7—C8	−0.3 (2)	N3—C15—C16—N4	−65.6 (2)
C15—N3—C7—C8	−171.34 (18)	C19—N4—C17—O2	176.5 (3)
C1—N1—C8—C7	1.2 (3)	C16—N4—C17—O2	4.4 (4)
C1—N1—C8—C9	−178.5 (2)	C19—N4—C17—O1	−4.0 (3)
N2—C7—C8—N1	−0.2 (3)	C16—N4—C17—O1	−176.09 (18)
N3—C7—C8—N1	179.50 (19)	C18—O1—C17—O2	−178.2 (2)
N2—C7—C8—C9	179.6 (2)	C18—O1—C17—N4	2.2 (3)
N3—C7—C8—C9	−0.7 (2)	C17—O1—C18—C19	0.2 (3)
N1—C8—C9—C10	1.9 (4)	C17—N4—C19—C18	3.8 (3)
C7—C8—C9—C10	−177.9 (2)	C16—N4—C19—C18	175.9 (2)
N1—C8—C9—C14	−178.8 (2)	O1—C18—C19—N4	−2.3 (3)
C7—C8—C9—C14	1.4 (2)		
